# A Longitudinal Analysis of the Impact of Digital Technologies on Sustainable Food Production and Consumption in the European Union

**DOI:** 10.3390/foods13081281

**Published:** 2024-04-22

**Authors:** Claudiu George Bocean

**Affiliations:** Department of Management, Marketing and Business Administration, Faculty of Economics and Business Administration, University of Craiova, 13 AI Cuza Street, 200585 Craiova, Romania; claudiu.bocean@edu.ucv.ro

**Keywords:** sustainable food production, sustainable food consumption, agricultural output, digital technologies, greenhouse gases, solid waste

## Abstract

In today’s landscape, digital technologies hold immense potential in tackling challenges associated with food sustainability. This study aims to contextualize a broader investigation of food sustainability and digitalization within the agricultural sector. Its objective is to explore the influence of digital technologies on sustainable food production and consumption, particularly examining relationships among digital technologies, municipal waste, agricultural output, nitrogen emissions, methane emissions from agriculture, and Goal 12 Responsible Consumption and Production (SDG12). Through the use of Structural Equation Modeling, the empirical investigation scrutinizes the relationships between digital technology use and critical variables linked to food sustainability in a longitudinal analysis. The results highlight the significant impact of extensive digital technology use on municipal waste, sustainable production, and consumption, indirectly influencing greenhouse gas (GHG) emissions. Empirical research findings reveal a negative influence of digital technologies on responsible consumption and production (path coefficient −0.349, *p* values < 0.001), suggesting an impact of digital technologies on diminishing sustainability in consumption and production. The relationship between digital technologies and municipal solid waste is also negative (path coefficient −0.360, *p* values < 0.001), suggesting that the use of digital technologies can contribute to reducing the amount of municipal solid waste. Digitalization has the potential to improve the sustainability of supply chains by reducing resource consumption and greenhouse gas emissions associated with production and distribution operations.

## 1. Introduction

Demographic changes, such as population growth and rapid urbanization, exert additional pressure on existing food and agricultural resources [1]. The increased demand for food requires sustainable approaches to ensure food security and protect the environment. Furthermore, climate change affects the availability and quality of natural resources essential for food production, emphasizing the need for adaptation and innovation in agriculture. These complex conditions call for integrated approaches and innovative solutions to ensure the sustainability of food systems and address future challenges. A complete transformation of agriculture is essential to address current challenges and ensure sustainable productivity growth. Evaluating and implementing new and advanced production technologies can play a crucial role in this transformation, including sustainable consumption patterns and GHG emissions [2,3].

Digital technologies can play a crucial role in addressing issues related to food sustainability. Digital technologies can assist in monitoring and efficiently managing food supply chains, thereby reducing food losses and waste. Automation can improve production process efficiency, contributing to resource consumption reduction and minimizing environmental impact. Biotechnologies can enhance crop quality, ensuring sustainable food production and resilience to climate change [4]. Therefore, investments in research and development of these technologies could bring significant benefits for promoting sustainable food systems worldwide.

The implementation of digital technologies in the supply chain, such as artificial intelligence (AI), Big Data (BD), Internet of Things (IoT), and cloud computing (CC), can enhance its transparency and visibility, allowing involved parties to monitor and manage the flow of goods and information more efficiently [5,6,7,8,9]. This enhancement can lead to better collaboration among supply chain partners and improve communication and coordination among them. Moreover, digital technologies can facilitate the implementation of sustainable practices in the supply chain, such as reducing carbon emissions and minimizing environmental impact through optimizing transportation routes and reducing waste [9].

Sustainable agriculture addresses environmental, social, and economic aspects with advanced technology improving efficiency and sustainability while reducing environmental impact and fostering economic opportunities [10]. Digital technologies offer solutions to optimize resource use, but enhancing understanding and acceptance of these tools is crucial for a sustainable transition [11,12,13,14].

This paper aims to explore the impact of digital technologies on food production and consumption with a specific focus on assessing their effects on municipal waste, which predominantly arises from food consumption, as well as nitrogen and methane emissions, primarily associated with agriculture. Furthermore, it examines their implications for sustainable consumption and production in alignment with Sustainable Development Goal 12 (SDG12) [9]. Identified research gaps encompass the absence of detailed longitudinal analyses regarding the impacts of digital technologies on food sustainability, encompassing municipal waste, agricultural GHG emissions, and SDG12. Moreover, a thorough exploration of the intricate interconnections among food production, consumption, and critical variables linked to agricultural sustainability is warranted.

The originality of this study resides in its utilization of Structural Equation Modeling to assess the correlations between the adoption of digital technologies and critical variables associated with overall and food sustainability. This research constitutes a significant contribution to comprehending how digital technologies can shape sustainable food production and consumption, mitigate food wastage, and furnish valuable insights for the development of policies and practices aimed at advancing food sustainability in the future.

The structure of this paper consists of six consecutive sections. The introduction presents the purpose and objectives of the research, while the literature review section and hypothesis development explore previous research and theoretical foundations. The materials and methods section describes the research methodology, and the results section presents the findings obtained. The discussion section analyzes the results and their implications, while the conclusions section summarizes the findings and contributions of the study.

## 2. The Literature Review

### 2.1. Digitalization in Agriculture

Sustainable agriculture must address not only environmental aspects but also social and economic ones. The appropriate use of advanced technology can improve the efficiency and sustainability of agricultural processes while simultaneously reducing environmental impact and creating economic opportunities for agricultural communities. Integrating ecosystem services and human capital into agricultural practices can promote the sustainable use of natural resources and contribute to increasing the resilience of agricultural systems to climate change and other challenges in producing food sustainably [10].

The issue of resource efficiency in the agri-food sector becomes increasingly persistent as the food needs of a growing population are accompanied by urbanization and changes in dietary habits [11]. Digital technologies offer promising solutions to optimize resource use in agriculture, but it is essential to improve understanding and acceptance of these technologies to ensure an efficient and sustainable transition to resource-efficient agricultural practices [12,13]. Education and access to suitable information and technological resources are critical elements in promoting the use of digital technologies for more sustainable and efficient agriculture [14].

Emerging AI, BD, and IoT can optimize agricultural processes, enable data-driven decision making, and effectively manage agricultural resources [15,16]. Concerning the production of high-protein foods, integrating digital technologies can improve the quality, yield, and sustainability of production processes. Optimizing protein contributions from animal and plant sources can help reduce excessive resource consumption and minimize environmental impact while promoting a balanced and healthy diet for global consumers [17].

Integrating digital technologies into agriculture offers significant opportunities for improving the performance and sustainability of the agricultural sector [18]. Digital technologies such as IoT and CC allow primary producers to monitor and manage crops and animals more efficiently, optimize resource use, and make more informed decisions. For example, IoT sensors can provide real-time information about soil and water quality and plant and animal health, enabling primary producers to respond more quickly to changes in environmental conditions and avoid resource waste [19]. Moreover, the use of digital technologies can help reduce the use of pesticides and chemical fertilizers, thereby reducing environmental pollution and risks to human health [20].

Precision agriculture can lead to more efficient use of resources and reduce environmental impact across the entire agri-food chain [21,22]. The potential benefits of these technologies depend on several factors, including the efficiency of implementation and the specific context of each region or agricultural sector [23]. Empirical studies are essential to assess the real impact of these technologies on the environment and to develop appropriate implementation strategies to maximize benefits and minimize adverse effects. Digital technologies are not a complete solution for sustainable agriculture, and integrated approaches that include agroecological principles are needed to ensure sustainable and environmentally friendly agricultural production [24].

Precision agriculture and innovative technologies can improve the efficient use of natural resources and reduce negative environmental impacts [25]. By utilizing these technologies, primary producers can apply agricultural inputs more precisely, thus reducing waste and pollution. For example, through the use of AI and IoT, primary producers can assess crop health and identify problem areas, allowing them to apply agricultural inputs only where necessary [26,27]. Moreover, IoT sensors can monitor soil moisture and other essential parameters, allowing primary producers to apply water and other inputs more efficiently, thereby reducing water consumption and avoiding over-irrigation [28].

Digital technologies enable more precise and efficient management of agricultural resources and processes. The development of agricultural databases and the use of data analysis tools can help primary producers collect and interpret information about soil conditions, weather, plant health, and other relevant factors, enabling them to make better-informed decisions [29]. These developments may include, for example, adjusting water and fertilizer applications based on the specific needs of crops and local climatic conditions, thereby reducing resource waste and environmental impact. Managing the interplay between soil, crop management techniques, and climatic factors can enhance agricultural productivity while reducing the likelihood of soil degradation and erosion [19]. Digital technologies can contribute to monitoring and reporting carbon emissions from the agricultural sector and implementing practices to reduce the impact of these emissions on climate change [20]. Therefore, integrating digital technology into agriculture has the potential to improve the sustainability and efficiency of the agricultural sector while also contributing to environmental protection and achieving climate change mitigation goals [21].

The European Union, through the Common Agricultural Policy and other initiatives, encourages the adoption of digital technologies in agriculture to enhance the efficiency, sustainability, and competitiveness of the European agricultural sector [30]. Collaboration among EU member states in digital agriculture demonstrates the shared desire and commitment to explore and harness the technological potential to address current and future challenges facing European agriculture. Through these collective efforts, the EU can play a crucial role in promoting more innovative, more sustainable, and resilient agriculture [21].

The implementation of digital technologies in sustainable food production and consumption brings forth a series of particularly significant economic implications. These technologies can revolutionize the entire food chain, from production to consumption, bringing considerable benefits but also challenges that require careful approaches and economic adaptability.

Regarding food production, digital technologies can improve efficiency and productivity in various agricultural sectors. The use of IoT (Internet of Things) sensors and monitoring technologies allows primary producers to collect real-time data on soil conditions, humidity, temperature, and other critical factors for plant and animal growth. This information can be used to optimize resource utilization, reduce waste, and increase yields, thereby contributing to the economic growth of farms.

Digital technologies implemented in agriculture can play a significant role in the economic advancement of developing countries [12,31]. By adopting innovative technological solutions, such as smart irrigation systems, using drones for crop monitoring, and digital platforms for access to agricultural information, these countries can improve the productivity and quality of agricultural production, thereby contributing to economic growth and poverty reduction in rural areas [32]. Digital technologies can improve primary producers’ access to markets, facilitate trade, and support the economic development of rural communities [33,34]. Online platforms and mobile applications facilitate access to information about food products, including origin, production process, and environmental impact [24]. Through these platforms, primary producers can receive real-time market information, including pricing data for their produce. By having access to such information, primary producers can make informed decisions about when and where to sell their products, ultimately leading to increased returns and improved economic viability [24,30]. Also, primary producers can receive payments directly to their mobile phones, bypassing the need for intermediaries and reducing transaction costs. This option not only improves the financial inclusion of small-scale primary producers but also enhances transparency and efficiency within the agricultural market [15]. These technologies can influence consumer purchasing preferences towards more sustainable and local products, creating increased demand for ecological and organic products [15,33].

However, the implementation of digital technologies in sustainable food production and consumption is not without economic challenges [18]. The initial costs for implementing these technologies can be high, and their adoption may require additional resources for training and personnel. There is a risk that these technologies may exacerbate economic disparities between large primary producers and small primary producers or local entrepreneurs who may have limited financial resources to invest in advanced technologies [9].

The implementation of digital technologies in sustainable food production and consumption has complex economic implications with a range of opportunities and challenges [10]. With a strategic approach and appropriate investments, these technologies can contribute to increasing the efficiency and sustainability of the entire food system while also providing long-term economic and social benefits [11].

Table 1 provides a comprehensive overview of the implementation of digital technologies, along with their associated benefits and obstacles, to facilitate a clearer understanding of the potential benefits and challenges of adopting digital technologies in agriculture and the agri-food system.

### 2.2. Sustainability and Innovation in the Food System

Projections regarding the world’s population growth and income rise indicate a significant increase in global food demand in the future [45]. By 2050, the world’s population is expected to grow by over 30% compared to the current population [4]. Globally, the consumption of meat and dairy products is projected to increase by 173% and 158%, respectively, between 2010 and 2050 [45]. This surge in demand puts considerable pressure on essential natural resources for food production, such as water, fertile land, and energy. Climate change poses additional risks to global food security, with potentially severe consequences for the environment and society [16,46].

The growing demand for high-quality and nutritious food exerts additional pressure on the global agri-food system, straining natural resources and ecosystems [47,48]. Agricultural expansion can lead to deforestation and the destruction of natural habitats, affecting biodiversity and contributing to the loss of vital ecosystem services [49]. Increased water consumption in agriculture can deplete freshwater resources and exacerbate water crises in different regions. GHG emissions from agriculture contribute to climate change and climatic instability, negatively impacting food production and food security [4].

The importance of the agri-food sector is highlighted by its extensive, diverse impact on our lives and economies. From agricultural production to food distribution and consumption, this sector plays a crucial role in providing food, supporting communities, and shaping cultural identity [50]. The massive engagement of the population in this sector underscores its importance and significant influence on labor market dynamics and socio-economic development [51]. In the context of climate change and other global challenges, ensuring sustainable agriculture and a food system becomes a crucial priority for promoting human well-being and protecting the environment. Wijerathna-Yapa and Pathirana [38] suggest attention and investment in developing and improving the agri-food sector to meet the demands and challenges of the future.

In light of increasing concerns about human health, consumers and policymakers are beginning to focus on alternative protein sources, helping reduce environmental pressure and promoting more equitable food systems [52]. For example, plant-based crops such as legumes and seeds require less water and land compared to animal meat production, and microalgae and insects can be provided with optimal growth conditions without requiring large amounts of land or water resources [53]. Thus, diversifying protein sources and promoting sustainable alternatives can contribute to better-balanced human diets and reduce negative environmental impacts [4]. However, there are also concerns and limitations with considering microalgae and insects as food sources. The cultivation of microalgae on a large scale may require significant infrastructure and energy inputs, potentially leading to environmental impacts such as habitat disruption and carbon emissions. Also, the acceptance of insects as a mainstream food source may face cultural and regulatory barriers in many regions [52]. Furthermore, there are potential risks related to allergenicity, food safety, and quality control that need to be addressed when incorporating these novel protein sources into human diets [53]. Ensuring the safety and sustainability of production processes is paramount to avoid unintended consequences and adverse health outcomes for consumers [4].

Table 2 offers a concise overview of both the positive impacts and challenges entailed by the integration of digital technologies within food supply chains, highlighting the transformative potential and hurdles within this domain [54,55,56,57,58,59,60,61,62,63,64,65,66,67,68,69,70,71,72,73,74,75,76,77,78,79,80,81,82,83,84,85,86,87,88].

Considering the findings of previous research, this paper proposes four hypotheses regarding the influences of digital technologies on sustainable production and consumption and waste resulting from human activity, including food waste, as seen below:

**Hypothesis** **1.** *The level of agricultural production has a positive influence on responsible consumption and production (SDG12).*

**Hypothesis** **2.** *Current digital technologies have a negative influence on responsible consumption and production (SDG12).*

**Hypothesis** **3.** *The level of agricultural production has a positive influence on municipal waste.*

**Hypothesis** **4.** *Current digital technologies have a negative influence on municipal waste.*

### 2.3. The Impact of the Agri-Food Chain on the Environment and GHG Emissions

The agreement on GHG emissions and the SDGs represents critical commitments of the international community in combating climate change and promoting sustainable development. These are essential tools for guiding policies and actions at the global, regional, and national levels towards a greener and more sustainable economy. By aligning strategies and efforts with these agreements and goals, governments, non-governmental organizations, and the private sector can collaborate towards a more prosperous and equitable future for all [89].

Agriculture significantly contributes to global emissions of GHG methane (CH_4_) and nitrous oxide (N_2_O), both through direct agricultural processes, such as animal enteric fermentation [90] and the application of chemical fertilizers [91], as well as through deforestation and land use changes associated with agriculture [92]. These emissions (approximately 12% of global GHG emissions) can have a significant impact on climate change, contributing to global warming and extreme weather phenomena [38]. Among greenhouse gases emitted by agriculture, methane (CH_4_) is the primary contributor, accounting for nearly 67% of emissions, followed by nitrous oxide (N_2_O) at 32% and carbon dioxide (CO_2_) at 1% [92].

Climate change caused by GHG emissions (CO_2_, nitrous oxide—N_2_O, methane—CH_4_, among the most significant) has severe and diverse consequences for the environment and life on Earth [93]. From rising sea levels and declining biodiversity to extreme weather events and impacts on agriculture and food security, the effects of global warming are already being felt worldwide and can have devastating consequences for future generations [94]. It is imperative to raise awareness and take action to reduce GHG emissions and adopt more sustainable practices to limit climate change and protect the environment for our future.

The impact of CH_4_ and N_2_O emissions on global warming is significant, and agriculture plays a significant role in this equation [90]. It is evident that agricultural practices, especially those in conventional agriculture, contribute to GHG emissions and, consequently, to climate change [95]. However, there are alternative solutions, such as organic farming, which can significantly reduce the impact of CH_4_ and N_2_O emissions [38]. Climate change has a significant impact on agri-food systems, causing changes in the availability and quality of natural resources, agricultural production, distribution, and access to food, as well as long-term impacts on food security and the sustainability of food systems. By adopting more sustainable agricultural practices and promoting environmentally friendly agricultural systems, we can help reduce the impact of agriculture on climate change and protect the environment for future generations.

Reducing GHG emissions in agriculture could contribute to mitigating the impact of climate change and improving the sustainability and efficiency of agricultural systems [96]. Implementing more ecological agricultural practices could also promote soil health, biodiversity, and food quality, benefiting society as a whole.

The significant increase in animal populations associated with CH_4_ emissions during enteric fermentation underscores the importance of taking concrete measures to reduce GHG emissions from the livestock sector [97]. Thus, adopting more sustainable practices on livestock farms as well as improving animal nutrition to reduce methane production [90] are necessary. Investments in innovative technologies for waste management and reducing the carbon footprint of meat production can significantly contribute to mitigating the impact of the livestock sector on climate change.

The increase in nitrogen usage in agriculture has led to significant improvements in crop production and global population sustenance in recent decades [92]. Efficient nitrogen utilization is crucial for achieving high yields and maintaining soil health. However, excessive or incorrect use of nitrogen fertilizers can have negative consequences on the environment, such as groundwater and surface water pollution as well as GHG emissions. Therefore, optimizing nitrogen use in agriculture is crucial for maintaining sustainable food production and a healthy environment.

Excessive use of agrochemicals can have severe consequences for the environment and human health [25]. Crop monitoring systems and precise irrigation management can reduce the dependence on agrochemicals and improve sustainable farming practices. Digital technologies can facilitate the exchange of information and best practices among primary producers and agricultural organizations, promoting a more conscious approach to agrochemical use and more efficient natural resource management. These efforts contribute to environmental protection and the long-term sustainability of agriculture in developing countries [31].

Animal husbandry, nitrogen-rich fertilizers, animal manure utilization, crop residue burning, and water management in flood-prone cultivation areas generate a variety of GHGs, including methane and nitrous oxide [90,92]. These agricultural practices can reduce associated GHG emissions. For example, modern agricultural technologies, such as efficient animal manure management and the use of organic fertilizers, can contribute to mitigating GHG emissions from agriculture. Moreover, regenerative agricultural practices, such as precision farming and organic farming, can help reduce the negative impact of agriculture on the climate and the environment. The use of digital technologies in agriculture can increase the efficiency, profitability, and sustainability of production [34]. When animal manure is not adequately managed or composted, pathogenic bacteria can contaminate the soil and crops. These bacteria can enter the food chain and cause severe illnesses in consumers [92]. Therefore, it is essential to pay special attention to the proper management of animal manure, including adequate composting and compliance with hygiene and food safety regulations and standards. These measures are crucial to prevent risks to public health and ensure that food products are safe for consumption [90].

Apart from operational benefits, digitalization can enhance the sustainability of supply chains by diminishing resource consumption and GHG emissions linked to production and distribution operations. The use of IoT technologies and data analysis can enable better monitoring of energy and water consumption in real time, allowing enterprises to identify and efficiently reduce resource consumption [54].

There are multiple investigations concerning the share of agriculture in global GHG emissions, but the impact on food production systems has been neglected [92]. This neglect has had significant consequences for the environment and our food system. Climate change has brought to the forefront the need to assess and improve the sustainability and efficiency of our agricultural systems. It is crucial to focus on reducing GHGs from agriculture and implementing more sustainable and environmentally friendly agricultural practices to mitigate environmental impact and adapt to ongoing climate changes.

Considering the previous research results, this paper proposes two hypotheses concerning the influences between sustainable production and consumption and GHG emissions from agriculture, as follows:

**Hypothesis** **5.** *Responsible consumption and production (SDG12) have a negative influence on the GHG emissions from agriculture.*

**Hypothesis** **6.** *The level of agricultural production has a significantly positive influence on the level of GHG emissions from agriculture.*

## 3. Materials and Methods

The research on the current influences of digital technologies on sustainable production and consumption models, considering food agricultural production, waste, and nitrogen and methane emissions from agriculture, has involved a process with five stages: the literature review, hypotheses formulation, data collection, data processing and hypotheses testing, presentation of results, discussions, and conclusions.

Data collected to investigate the relationships between current digital technologies, SDG12, crop and animal output, municipal waste, and nitrogen and methane emissions from agriculture come from three sources: Digital Economy and Society Index (DESI) reports, Sustainable Development Report 2023, and Eurostat database.

From the European Digital Society Index (DESI) report, this paper retained two dimensions to illustrate digital technologies: Integration of Digital Technology (IDT) and Digital Public Services (DPSs). These two dimensions showed significant external loadings and external weights statistically (*p* < 0.05) in the tested SEM model. The other two dimensions, Human Capital (HC) and Internet Connectivity (C), considered in the initial model were not included in the final model as a result of insignificant outer weights (*p* > 0.05). Integration of Digital Technology assesses the degree of digital technology integration, including the use of digital technologies in business processes, production, and innovation. Digital Public Services assess the degree of digitalization of public services provided by public authorities, such as e-governance systems, online processes for obtaining official documents, and other digitally available public services.

From the Sustainable Development Report 2023, this paper selected two critical aspects: SDG12—Sustainable Consumption and Production and SDG12_msw—Municipal solid waste. SDG12 measures progress towards sustainable production and consumption, including the use of natural resources, waste management, and reducing environmental impact. SDG12_msw focuses on the management of municipal solid waste, including food waste. By evaluating this indicator, a better understanding of solid waste management in society and the environmental and public health impacts can be achieved. Analyzing this aspect can help identify more efficient strategies for managing and reducing solid waste, including food waste. Both indicators address goods and services in general, not only food products.

Data selected from the Eurostat database included crop and animal output, methane emissions (expressed in CO_2_ equivalent), and nitrous oxide emissions (expressed in CO_2_ equivalent) from agricultural activities, hunting, and related services.

The investigation included all selected indicators from the period 2017–2022. The longitudinal analysis undertaken over six years (2017–2022) is helpful in investigating trends and changes over time regarding the impact of digitalization on production and consumption. We selected this timeframe because it provided the most robust and comparable data sets aligned with the objectives of our research. The data collected during this period allowed for a comprehensive examination of the evolving dynamics within the digital economy. Table 3 presents the variables used in the investigation.

**Figure 1:**
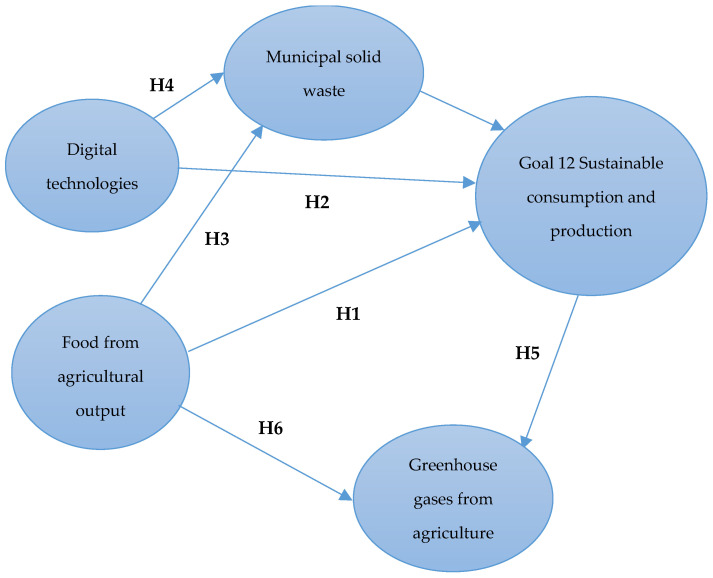
Theoretical model. Source: Developed by the author.

Theoretical modeling of the research illustrates the connections between the model variables regarding the current impact of digital technologies on sustainable production and consumption, considering food agricultural output, waste, and nitrogen and methane emissions from agriculture (Figure 1).

This paper uses Structural Equation Modeling (SEM) as the primary analytical tool due to its capacity to examine the complex relationships among variables within a theoretical model [102]. SEM enables the simultaneous evaluation of multiple variables and their interdependencies, making it suitable for studying the multidimensional dynamics of digital technology’s impact on sustainable production and consumption, as well as GHGs in agriculture [103]. Previous research in the field of agriculture has successfully employed SEM in investigating influences among heterogeneous variables [104,105,106].

SEM provides several advantages in this context. Firstly, it allows for testing hypotheses derived from the theoretical model, enabling researchers to assess the direct and indirect effects of digital technologies on various aspects of sustainable production and consumption [105]. Secondly, SEM enables the integration of multiple data sources, such as DESI reports, Sustainable Development Reports, and the Eurostat database [107]. SEM provides statistical measures of model fit, assisting researchers in globally evaluating the validity and reliability of the proposed theoretical framework [102].

By utilizing SEM and integrating diverse data sources, the research aims to provide a comprehensive analysis of the impact of digital technologies on sustainable food systems. Through empirical analysis and hypothesis testing, the study seeks to uncover insights that can inform policies and practices in promoting more efficient and sustainable food production and consumption practices, which are in line with SDG12.

## 4. Results

**Figure 2:**
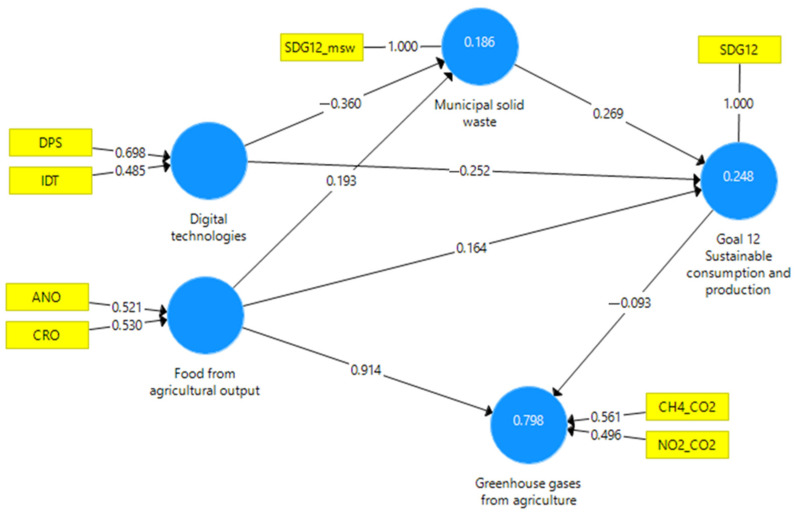
PLSc model. Source: Developed by the author using SmartPLS v3.0.

The SEM model chosen was formative tested with the consistent PLS (Partial Least Squares) algorithm within SmartPLS v.3.0 [108]. Several researchers advocate for the use of a consistent PLS algorithm (PLSc) to enhance the understanding of structural relationships [103,109,110]. The latent variables of the model were digital technologies, food from agricultural output, Goal 12 sustainable consumption and production, GHGs from agriculture, and municipal solid waste. Figure 2 illustrates the model obtained after applying the PLSc.

In the case of formative models, the determination of the variables’ collinearity performed by examining the VIF (Variance Inflation Factor) values of formative indicators is essential [107]. For the PLSc model, the calculated VIF values are below the threshold value of five (Table 4). Consequently, collinearity does not reach critical levels and does not pose a problem for estimating the PLSc path model.

Subsequently, it is necessary to analyze outer weights to observe the significance and relevance of the formative PLSc model [107]. The bootstrapping technique enables the determination of the significance of outer weights. For the PLSc model, we applied a bootstrapping procedure considering 10,000 samples with a 0.05 significance level, using two-tailed testing. Table 5 presents the outer weight results for the exogenous variables of the model.

Model fit was also verified. The indicators illustrating model fit record significant values: SRMR < 0.08 and NFI > 0.9 [102]. Table 6 describes model fit indicators.

Due to the bootstrapping procedure, we calculated the path coefficients of the PLSc model, as illustrated in Table 7.

**Figure A1:**
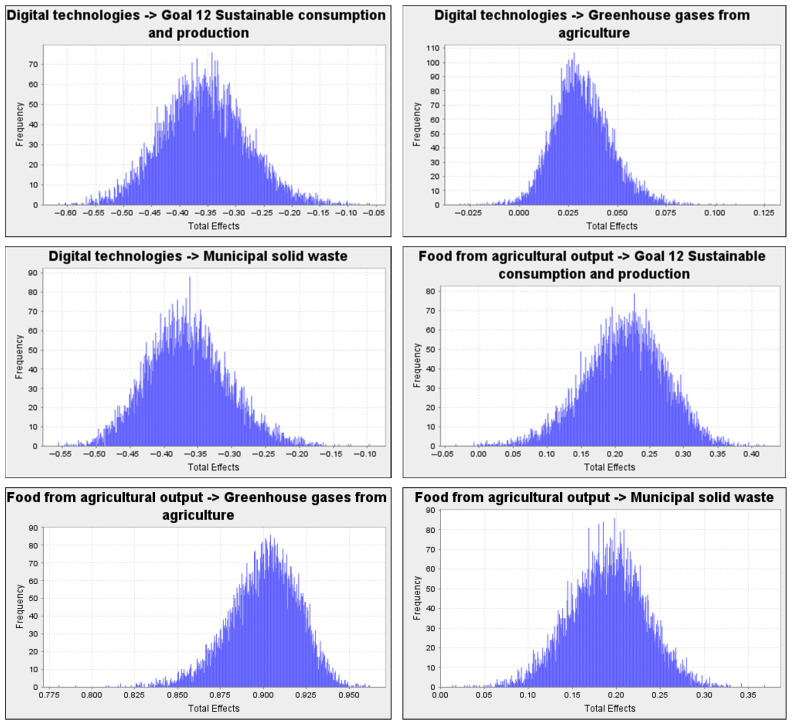

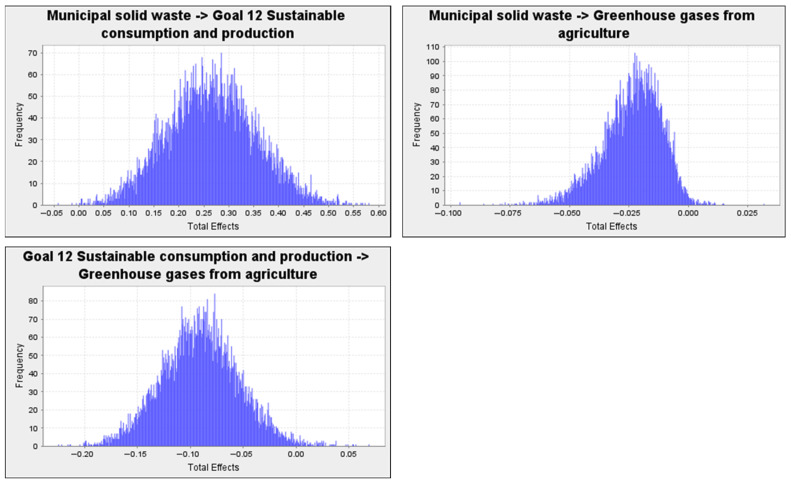
Histograms for total effects. Developed by the author based on data using SmartPLS v3.0.

Investigating proposed hypotheses involves testing both direct and indirect effects. Therefore, during the bootstrapping procedure, we calculated the total effects. Table 8 presents the total effects recorded between the formative PLSc model variables, while Figure A1 presents histograms showing the dispersion of estimated values across iterations for total effects.

The path coefficients obtained through bootstrapping in the consistent PLS model reflect the total effects between the variables considered in the study and provide important information about their reciprocal influences. The results reveal a negative influence of digital technologies on responsible consumption and production (SDG12), suggesting an impact of digital technologies on diminishing sustainability in consumption and production. This outcome supports the H2 hypothesis. Agricultural production demonstrates a notable positive influence both on sustainable consumption and production (SDG12) and municipal solid waste. This finding underscores a robust link between agricultural production and sustainability, alongside a potential rise in municipal solid waste with escalating agricultural production. These results support the H1 and H3 hypotheses. Furthermore, the relationship between digital technologies and municipal solid waste is also negative, suggesting that the use of digital technologies can contribute to reducing the amount of municipal solid waste, validating the H4 hypothesis.

Regarding the total effects between SDG12 and GHGs from agriculture, the path coefficient demonstrates a negative value, implying a detrimental impact of sustainable consumption and production on GHG emissions from agriculture, validating the H5 hypothesis. The relationship between the level of agricultural production and the level of GHG emissions of nitrogen and methane from agriculture is positive, suggesting that an increase in crop and animal output will lead to an increase in GHG emissions. This result validates the H6 hypothesis.

## 5. Discussion

The use of technology in agriculture can have a significant impact on the efficiency and sustainability of food systems. However, for technology to have a transformative impact, it must be adequately implemented and take into account the specific social, economic, and cultural contexts of each community [40,111,112,113,114]. Improving access to food and reducing the carbon footprint of the global agri-food system are critical priorities for achieving sustainable development goals and ensuring global food security [115,116].

The objective of this study was to explore the impact of digital technologies on food production and consumption, with a specific focus on examining their influence on various aspects, including municipal waste primarily originating from food consumption, nitrogen and methane emissions from agriculture, and sustainable consumption and production in alignment with SDG12. The study used longitudinal data to achieve this objective, examining trends and changes over time regarding the impact of digital technologies on sustainable food production and consumption. Furthermore, the study investigated the relationships between agricultural production, municipal waste, and GHG emissions from agriculture in the context of sustainable development goals to gain a deeper understanding of the complex interactions in the food sector.

The investigation revealed a negative relationship between digital technologies and SDG12, suggesting that the impact of these technologies on sustainable consumption and production is moderate but significant. This fact suggests that the utilization of digital technologies might have a detrimental impact on sustainable development goals, raising concerns about sustainability in consumption and production and validating the H2 hypothesis. These findings emphasize the necessity for a more cautious approach when implementing digital technologies within the realm of sustainable food practices. Similarly, Lajoie-O’Malley et al. [117] showed that implementing digital technologies in agriculture can have certain risks and may not always bring the expected benefits. Increased efficiency can paradoxically lead to increased resource use. There is a risk that these technologies may accentuate inequalities and create new digital divisions, especially if small primary producers do not have access to or cannot benefit from them for various reasons or may have adverse effects on GHG emissions due to high energy consumption. Likewise, Kamble et al. [118] underscored that digitalization also presents challenges, including the imperative to guarantee equitable access to technology and data for all stakeholders engaged in the food supply chain. Additionally, there is a need to tackle issues related to data protection and cybersecurity within an ever more intricate and interconnected digital landscape. Bahn et al. [40] showed that digital technologies bring real and lasting benefits to the entire agricultural sector.

In contrast, this paper found that agricultural production has a significant positive influence on both sustainable consumption and production (SDG12) and the generation of municipal solid waste. This finding highlights the close connection between agricultural production and the concept of sustainability, suggesting that increasing agricultural production can bring both benefits and challenges in terms of municipal waste management. These results are in line with the H1 and H3 hypotheses, emphasizing the complexity of the relationship between agricultural production and food sustainability. Consistent with these findings, Wijerathna-Yapa and Pathirana [36] showed that agriculture is a pillar of economies and societies with a significant impact on food security, employment, and economic development. Thus, investments in modernizing and digitalizing the agricultural sector can contribute to increasing productivity, improving food safety standards, and enhancing competitiveness in the global market [55,57].

The research results revealed a negative relationship between digital technologies and municipal solid waste, indicating that the use of digital technologies can mitigate municipal solid waste, validating the H4 hypothesis. This finding suggests digital technologies could have a positive impact on solid waste management, thus contributing to greater sustainability of the food system. The research findings align with the findings of Bahn et al. [41], who suggested that digital technologies implemented in food systems can contribute to reducing waste by optimizing production, distribution, and inventory management processes [59]. These technologies can enable more efficient monitoring and management of the food chain, thereby reducing losses during transportation and storage and improving data management for production and distribution planning [19]. However, it is essential to consider the indirect impact of digital technology use on the environment, including additional energy consumption and the generation of electronic waste, to ensure a holistic and sustainable approach to the use of these technologies in agriculture and the food chain [119].

Regarding the total effects of SDG12 on GHG emissions from agriculture, we found that sustainable consumption and production have a negative influence on these emissions, validating the H5 hypothesis. This finding suggests that sustainable development goals could contribute to reducing GHG emissions from agriculture. This result underscores the importance of more sustainable consumption and production in reducing environmental impact. Consistent with these findings, Dong et al. [54], Agrawal et al. [120], and Sharma et al. [74] emphasize that companies can improve their operations by efficiently using digital technologies while simultaneously contributing to the global effort for a more sustainable future by achieving SDGs, especially SDG 12. The use of digital technologies can enhance operational efficiency, reduce GHG emissions [31,93], and contribute to strengthening supply chain sustainability [121]. Digital technologies can increase operational efficiency while reducing environmental impact [122]. Furthermore, the digital technologies used in supply chains can enhance transparency and accountability throughout the process, facilitating monitoring and reporting of sustainable practices [59].

Lastly, the positive relationship between agricultural production levels and GHG emissions from agriculture confirms the validity of the H6 hypothesis. This result suggests that an increase in agricultural production may contribute to an increase in GHG emissions, highlighting the need for improved agricultural practices to reduce environmental impact. As shown by Kabange et al. [90], Xu et al. [91], and Wang and Ouyang [34], agriculture constitutes a substantial source of global GHG emissions, stemming from direct agricultural activities like animal fermentation and the application of chemical fertilizers. These emissions contribute to climate change and environmental degradation. However, implementing improved agricultural practices and modern technologies can help reduce these emissions and promote more sustainable agriculture [92]. Digital technologies can enhance the efficiency, profitability, and sustainability of agricultural production, providing primary producers with essential tools and real-time decision-making information [34].

The result of this hypothesis examination leads us to identify appropriate policies and practices in the food sector under more efficient use of digital technologies in agriculture to meet SDGs. Implementing digital technologies into the agricultural and food sectors can yield substantial advantages, enhance efficiency, provide access to information, and foster innovation across the entire food supply chain [12]. However, additional efforts are needed to promote the adoption of digital technologies by all stakeholders and to facilitate access to these technologies, especially for small- and medium-sized primary producers [14]. Investments in digital infrastructure and digital education can play a crucial role in supporting the transition to a more efficient and sustainable agri-food system [11].

The integration of digital technologies in agriculture should support sustainability and resilience objectives in the agricultural sector. In this context, the EU is adopting a proactive stance to promote digitalization in agriculture as a component of transitioning towards more sustainable and efficient agricultural systems. The concept of the fourth agricultural revolution highlights the importance and potential of digital technology to radically transform the way agriculture is managed and practiced in the future [123]. Through digitalization, production processes, resource monitoring, and decision making can be improved, contributing to increased efficiency and sustainability in agriculture. The EU can act as a role model for other regions and countries, showcasing how digitalization fosters more environmentally friendly and adaptable agriculture in response to present and future challenges [21].

Digital technologies used in agriculture can bring multiple benefits, such as increased operational efficiency, reduced negative environmental impact, improved food quality and safety, and increased primary producers’ incomes [114]. These technologies can support the adoption of more sustainable agricultural practices and contribute to reducing dependence on external inputs, such as pesticides and chemical fertilizers, while promoting the more efficient use of natural resources [124]. Furthermore, they can facilitate access to agricultural information and services for primary producers in rural areas and enhance transparency and efficiency throughout the entire food chain [125].

Digitalizing the food system presents numerous advantages and opportunities for improving efficiency, transparency, and sustainability throughout the entire food supply chain [126,127]. Digital technologies enable risk identification and management more efficiently, optimizing the food production and distribution process, and new business models can contribute to achieving SDGs, particularly in terms of eliminating hunger, promoting health and well-being, combating climate change, and promoting sustainable patterns of production and consumption [128,129].

However, the high cost of advanced agricultural technologies can be a significant obstacle for primary producers and other entities in the agricultural sector, mainly in developing countries. Furthermore, lack of training and access to resources necessary for learning and implementing these technologies may limit their widespread adoption, create digital divides, and accentuate inequalities [118].

### 5.1. Theoretical Implications

The challenges facing food systems, such as population growth, competition for resources, the complexity of the global food chain, dietary patterns, climate change, limited food access, unsustainable agricultural practices, and food waste, reflect the complexity and fragility of current food systems. Population growth and rapid urbanization create additional pressures on existing natural and agricultural resources, accentuating risks of food insecurity and environmental degradation. Climate change exacerbates these issues, affecting agricultural production and access to food for millions of people worldwide. Unsustainable agricultural practices underscore the need for structural reforms in agriculture and food supply chains to promote more sustainable and equitable production and consumption. Reducing food waste and improving access to nutritious and sustainable food are critical priorities for achieving food security and sustainable development in the future.

A sustainable food system is crucial not only for human health but also for the well-being and sustainability of the environment and economies. A food system optimized for sustainability can have long-term benefits for human health, ensuring access to healthy and safe food while simultaneously protecting the environment and supporting agricultural and rural communities.

### 5.2. Practical Implications

Agriculture is an essential component of economies and livelihoods worldwide, providing food and other necessary products for daily life. This paper highlights the need for the development of sustainable and efficient agricultural practices to reduce the negative impact of the agricultural sector on the environment. The current model of production and consumption reveals imbalances in current food production and consumption systems that contribute to exacerbating the impact of climate change by increasing GHG emissions. The findings of the empirical study emphasize the urgent need to develop and implement more sustainable and environmentally friendly agricultural practices that reduce the agricultural sector’s impact on climate change and contribute to protecting the environment for future generations.

This paper underscores the potential of these technologies to optimize resource utilization and mitigate environmental impacts, thereby promoting more sustainable consumption and production patterns. Moreover, it emphasizes their role in reducing municipal waste through improved resource management practices. However, these technologies have potential side effects, such as resource overuse, that may occur in the absence of adequate regulations and sustainable resource management strategies. A balanced approach is needed to ensure that digital technologies are used responsibly and sustainably within agricultural and food systems.

The development and promotion of digital technologies require an appropriate legislative and policy framework that keeps pace with the rapid rate of technological innovation and facilitates their adoption throughout agriculture, not just the food industry. Furthermore, a greater understanding of primary producers’ perspectives and needs regarding the use of digital technologies is necessary to develop solutions that better meet their requirements and support a more efficient and inclusive digital transformation in agriculture.

### 5.3. Limitations and Further Research

Despite efforts to investigate the relationships between digital technologies, sustainable production and consumption, food agricultural production, GHG emissions, and municipal waste, this paper has certain limitations. One of these limitations arises from the longitudinal nature of the analysis. Although we attempted to track the evolution of relationships over an extended period, contextual factors or unforeseen events may have influenced the results and interpretations. The study predominantly focused on the relationship between digital technologies, crop and animal output, and critical variables associated with food sustainability, such as municipal waste, nitrogen and methane emissions, and SDG12. However, other relevant aspects of food sustainability could be considered, including the social and economic impact of technological transformations in agriculture.

Future studies could investigate how digital technologies influence aspects such as agricultural labor employment, market access for small primary producers, or the equitable distribution of technology benefits. Another research direction should focus on exploring the unintended consequences of digitalization in agriculture. It is crucial to evaluate not only the positive aspects but also the potential adverse effects of digitalization in agriculture, such as the risk of digital exclusion or the concentration of economic power in the hands of large corporations. Future studies could investigate the specific effects of different digital technologies, such as the IoT, data analytics, or AI, on food sustainability.

## 6. Conclusions

Using technology in agriculture can have a significant impact on the efficiency and sustainability of food systems. However, for technology to have a transformative impact, it must be adequately implemented and take into account the specific social, economic, and cultural contexts of each community. It is essential to understand how technological solutions can address local needs and priorities and promote sustainable and inclusive agriculture. By researching and identifying elements that can facilitate the transformation of food systems through systemic innovations, more efficient policies and practices can be developed to address current and future challenges of food security and sustainable development.

Despite sufficient food resources, access to food remains a significant issue for millions of people worldwide. Improving access to food and reducing the carbon footprint are critical priorities for achieving SDGs and ensuring food security. The integration of digital technologies in agri-food chains can bring multiple benefits, but efforts are needed to promote their widespread adoption, ensuring they are accessible and beneficial to all stakeholders in the agri-food sector. By investing in digital infrastructure, education, and appropriate policies, countries can contribute to achieving a more efficient, sustainable, and inclusive food system that meets the current and future needs of society.

## Figures and Tables

**Table 1:** Benefits and obstacles of digital technology implementation.

Digital Technology Implementation	Benefits	Obstacles
Smart irrigation systems, drones for crop monitoring, and digital platforms for access to agricultural information [31,32]	- Improved productivity and quality of agricultural production; - Economic growth and poverty reduction in rural areas; - Enhanced access to markets and trade facilitation.	- Initial costs of implementing digital technologies; - Technical complexity of adoption.
IoT, AI, drones, and autonomous vehicles [35,36]	- More efficient monitoring and management of crops and livestock; - Optimization of resource utilization; - Data-driven decision making; - Timely preventive measures; - Enhanced efficiency and sustainability of agricultural production; - Contribution to food security and resilience to future challenges.	- Data security concerns; - Initial investment costs; - Technical capabilities and infrastructure limitations.
Integration of digital technologies into the agri-food system [37,38]	- Process optimization and cost reduction; - Improved efficiency across the food supply chain; - Transparent and safe supply chain management; - Promotion of socially and environmentally sustainable practices; - Increased competitiveness and resilience of the food system.	- Data privacy concerns; - Technical complexity of integration; - Training and adoption challenges.
Adoption of digital technologies during the COVID-19 pandemic [38,39,40]	- Enhanced resilience of the food supply chain during crises; - Increased interest and investment in digital solutions.	- Risks related to data security; - Costs associated with implementation; - Technical expertise requirements.
Integrated digital technologies in agriculture [34,41,42,43,44]	- Production optimization and resource consumption reduction; - Improvement in product quality and process efficiency; - Increased operational efficiency; - Enhanced monitoring and management of natural resources; - Support for efficient and sustainable production; - Informed and precise decision making; - Cost reduction; - Minimized environmental impact; - Enhanced access to information and educational resources.	- Initial costs and technical complexity of adoption; - Resistance to change within the industry; - Skills gap and training needs for primary producers.

Source: Developed by the author based on [31,32,33,34,35,36,37,38,39,40,41,42,43,44].

**Table 2:** Positive contribution and challenges of digital technology implementation in food supply chains.

Benefits	Challenges
- Reduced food waste and improved sustainability across the entire food system [54]	- Digital polarization and unequal access to technology [78]
- Sustainable production practices contribute to conserving natural resources and protecting biodiversity [55,56,57,58,77]	- Increased GHG emissions due to higher energy demand [68]
- Long-term economic, social, and ecological benefits from investments in improving efficiency and sustainability of the food supply chain [2,3,55,56,57,58,59,82,83,84,85]	- Risk of worsening social issues such as unemployment among unskilled workers [78,79]
- Better resource planning, coordination, and optimization through supply chain management systems [59,62,63,64,65,66,67,68,86,87,88]	- Excessive dependence on technology at the expense of traditional and sustainable approaches [78]
- Improved efficiency and sustainability of operations through the application of digital technologies [9,60,69,70,71,72,73,74,75,76]	- Addressing ethical and regulatory considerations linked to the adoption of digital technologies [80,81]
- Improved efficiency and transparency in managing food stocks, monitoring the supply chain, and optimizing transportation routes [9,54,61]	- Expensive implementation costs [40]

Source: Developed by the author based on [2,3,9,54,55,56,57,58,59,60,61,62,63,64,65,66,67,68,69,70,71,72,73,74,75,76,77,78,79,80,81,82,83,84,85,86,87,88].

**Table 3:** Variables and measures.

Variables	Data	Measures	Sources
DPSs	Digital Public Services	Score	[98]
IDT	Integration of Digital Technology	Score	[98]
CRO	Crop output	Million purchasing power standards (PPS)	[99]
ANO	Animal output	Million purchasing power standards (PPS)	[99]
SDG12	Sustainable consumption and production	Score	[100]
SDG12_msw	Municipal solid waste (kg/capita/day)	kg/capita/day	[100]
CH_4__CO_2_	Methane (CO_2_ equivalent)	Thousand tonnes	[101]
NO_2__CO_2_	Nitrous oxide (CO_2_ equivalent)	Thousand tonnes	[101]

Source: Developed by the author based on [98,99,100,101].

**Table 4:** Assessing multicollinearity.

	VIF
ANO	2.921
CRO	2.921
DPS	1.203
IDT	1.203
CH_4__CO_2_	2.676
NO_2__CO_2_	2.676
SDG12	1.000
SDG12_msw	1.000

Source: Developed by the author based on data using SmartPLS v3.0.

**Table 5:** Outer weights.

	Original Sample	Sample Mean	Standard Deviation	T Statistics	*p* Value Significance
ANO → Food from agricultural output	0.521	0.531	0.148	3.526	0.000 < 0.05
CRO → Food from agricultural output	0.530	0.516	0.151	3.514	0.000 < 0.05
DPS → Digital technologies	0.698	0.688	0.140	4.974	0.000 < 0.05
IDT → Digital technologies	0.485	0.480	0.159	3.041	0.002 < 0.05
CH_4__CO_2_ → Greenhouse gases from agriculture	0.561	0.564	0.162	3.455	0.001 < 0.05
NO_2__CO_2_ → Greenhouse gases from agriculture	0.496	0.488	0.168	2.955	0.003 < 0.05
SDG12 → Goal 12 Sustainable consumption and production	1 000	1 000	0.000		
SDG12_msw → Municipal solid waste	1 000	1 000	0.000		

Source: Developed by the author based on data using SmartPLS v3.0.

**Table 6:** Model fit.

	Saturated Model	Estimated Model
SRMR	0.032 < 0.08	0.033 < 0.08
d_ULS	0.037	0.040
d_G	0.036	0.038
Chi-Square	28.402	29.877
NFI	0.961 > 0.9	0.959 > 0.9

Source: Developed by the author based on data using SmartPLS v3.0.

**Table 7:** Path coefficients.

	Original Sample	Sample Mean	Standard Deviation	T Statistics	*p* Value Significance
Digital technologies → Goal 12 Sustainable consumption and production	−0.252	−0.261	0.074	3.386	0.001 < 0.05
Digital technologies → Municipal solid waste	−0.360	−0.366	0.059	6.064	0.000 < 0.05
Food from agricultural output → Goal 12 Sustainable consumption and production	0.164	0.162	0.057	2.898	0.004 < 0.05
Food from agricultural output → Greenhouse gases from agriculture	0.914	0.920	0.019	48.872	0.000 < 0.05
Food from agricultural output → Municipal solid waste	0.193	0.190	0.043	4.493	0.000 < 0.05
Goal 12 Sustainable consumption and production → Greenhouse gases from agriculture	−0.093	−0.091	0.037	2.538	0.011 < 0.05
Municipal solid waste → Goal 12 Sustainable consumption and production	0.269	0.266	0.089	3.004	0.003 < 0.05

Source: Developed by the author based on data using SmartPLS v3.0.

**Table 8:** Total effects.

	Original Sample	Sample Mean	Standard Deviation	T Statistics	*p* Values
Digital technologies → Goal 12 Sustainable consumption and production	−0.349	−0.357	0.075	4.621	0.000 < 0.05
Digital technologies → Municipal solid waste	−0.360	−0.366	0.059	6.064	0.000 < 0.05
Digital technologies → Greenhouse gases from agriculture	0.032	0.032	0.015	2.209	0.027 < 0.05
Food from agricultural output → Goal 12 Sustainable consumption and production	0.216	0.213	0.059	3.644	0.000 < 0.05
Food from agricultural output → Greenhouse gases from agriculture	0.893	0.900	0.020	44.155	0.000 < 0.05
Food from agricultural output → Municipal solid waste	0.193	0.190	0.043	4.493	0.000 < 0.05
Goal 12 Sustainable consumption and production → Greenhouse gases from agriculture	−0.093	−0.091	0.037	2.538	0.011 < 0.05
Municipal solid waste → Goal 12 Sustainable consumption and production	0.269	0.266	0.089	3.004	0.003 < 0.05
Municipal solid waste → Greenhouse gases from agriculture	−0.025	−0.024	0.013	1.996	0.046 < 0.05

Source: Developed by the author based on data using SmartPLS v3.0.

## Data Availability

Research data are publicly available: https://digital-strategy.ec.europa.eu/en/library/digital-economy-and-society-index-desi-2017 (accessed on 22 February 2024); https://digital-strategy.ec.europa.eu/en/library/digital-economy-and-society-index-2018-report (accessed on 22 February 2024); https://digital-strategy.ec.europa.eu/en/library/digital-economy-and-society-index-desi-2019 (accessed on 22 February 2024); https://digital-strategy.ec.europa.eu/en/library/digital-economy-and-society-index-desi-2020 (accessed on 22 February 2024); https://digital-strategy.ec.europa.eu/en/library/digital-economy-and-society-index-desi-2021 (accessed on 22 February 2024); https://digital-strategy.ec.europa.eu/en/library/digital-economy-and-society-index-desi-2022 (accessed on 22 February 2024); https://dashboards.sdgindex.org/static/downloads/files/SDR2023-data.xlsx (accessed on 18 April 2024); https://ec.europa.eu/eurostat/databrowser/view/env_ac_ainah_r2__custom_10475061/default/table?lang=en (accessed on 6 March 2024); https://ec.europa.eu/eurostat/databrowser/view/aact_eaa07__custom_10475106/default/table?lang=en (accessed on 5 March 2024).
